# Effect of Very High Charge Density and Monomer Constitution on the Synthesis and Properties of Cationic Polyelectrolytes

**DOI:** 10.3390/polym8060234

**Published:** 2016-06-16

**Authors:** Hamideh Ahmadloo, Ricardo Losada, Christine Wandrey

**Affiliations:** Institut d’Ingénierie Biologique et Institut des Sciences et Ingénierie Chimiques, Ecole Polytechnique Fédérale de Lausanne, station 15, Lausanne CH-1015, Switzerland; hamideh.ahmadloo@gmail.com (H.A.); rlm@teknologisk.dk (R.L.)

**Keywords:** free-radical polymerization, ionic monomers, water soluble polymers, polyelectrolytes, counterion condensation, autoacceleration

## Abstract

The free-radical homopolymerization of 1,3-bis(*N,N,N*-trimethylammonium)-2-propylmethacrylate dichloride (di-M) and 1,3-bis(*N,N,N*-trimethylammonium)-2-propylacrylate dichloride (di-A) in aqueous solution yields cationic polyelectrolytes (PEL) with theoretical/structural charge spacing of only ≈0.12 nm. The high charge density causes condensation of ≈82% of the chloride counterions. The high level of counterion condensation reduces the ionic strength in the polymerizing batch when the monomer molecules connect to PEL chains. This has the consequence that the hydrodynamic and excluded volume of the PEL molecules will change. Studies of the free radical polymerization revealed non-ideal polymerization kinetics already at low conversion and additionally autoacceleration above a certain monomer concentration and conversion. Similar autoacceleration was not observed for monomers yielding PEL with charge spacing of 0.25 or 0.5 nm. Coulomb interactions, monomer association, steric effects, and specific features of the monomer constitution have been evaluated concerning their contributions to the concentration dependence and conversion dependence of kinetic parameters. The different backbone constitutions of di-M and di-A not only influence the polymerization kinetics but also equip poly(di-M) with higher hydrolytic stability. The experimental results confirm the impact of electrochemical parameters and the necessity to reconsider their inclusion in kinetic models.

## 1. Introduction

Polyelectrolytes (PEL) can be produced either by chemical modification of neutral macromolecules or by polymerization of ionic/charged monomers [1]. The free-radical solution polymerization of charged monomers follows the usual mechanism of radical chain polymerization. However, it is known and generally accepted that the polymerization of ionic monomers in aqueous solution is affected by electrostatic forces/Coulomb interactions. During the polymerization of ionic monomers, these monomers do not only act as monomer molecules but also as a low molar mass electrolyte. As reported for diallyldimethylammonium chloride (DADMAC) [2] and other cationic monomers [1,3], the charge density has a strong impact on the polymerization behavior. Interaction of the PEL backbone charges with counterions, repulsion between the charges at the polymer backbone, and repulsion between the charge at the growing polymer radical and the ionic monomer, are all influenced by the ionic strength and can cause significant deviation from ideal polymerization behavior. As an example, kinetic studies on DADMAC within the commercially interesting range of monomer concentration, higher than 1 mol/L, revealed significant deviation from the ideal kinetic scheme. The exponential increase of the reaction rate with the monomer concentration was assigned to linear enhancement of the ratio of the propagation to the termination rate coefficient, *k_p_/k_t_*^0.5^, toward higher DADMAC concentration [2].

Despite the fact that many authors and abundant patents have described the synthesis, properties, and applications of a large variety of homo- and copolymers of ionic monomers, few ionic monomers have been the subject of detailed kinetic-mechanistic studies in aqueous medium [4]. This holds in particular for the homopolymerization of permanently charged ionic monomers yielding PEL, whose high charge density causes counterion condensation. For permanently charged PEL, the extent of counterion condensation is almost independent of the pH. Besides, not much attention has been paid to the variation of the ionic strength in the polymerizing batch. The ionic strength decreases when the fully dissociated ionic monomer molecules form PEL chains on which previously free counterions condense.

Several studies focused on the free-radical polymerization (FRP) of non-permanently charged monomers such as acrylic or methacrylic acid, which undergo ionization upon changing the pH [5,6,7,8,9,10,11,12]. In the case of ionized acrylic and methacrylic acid, complex influences of the monomer constitution and monomer concentration on the kinetics have been suggested [13,14,15,16]. Other studies reported on the polymerization of sulfonic acid derivatives [17,18]. Importantly, all previous kinetic-mechanistic polymerization studies used monomers, which yielded PEL with theoretical/structural charge spacing of ≥0.25 nm.

**Scheme 1:**
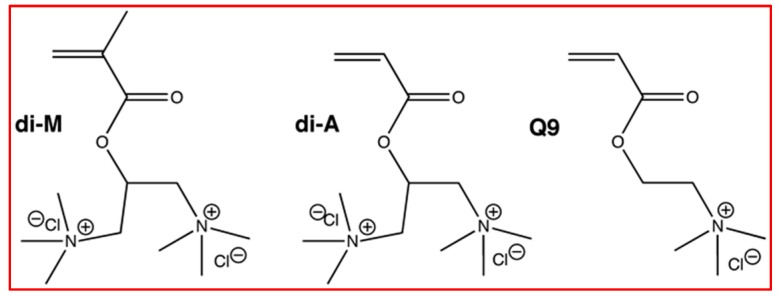
Chemical structures of the double-charged cationic monomers 1,3-bis(*N,N,N*-trimethylammonium)-2-propylmethacrylate dichloride (di-M) and 1,3-bis(*N,N,N*-trimethylammonium)-2-propylacrylate dichloride (di-A), and of the mono-charged cationic monomer acryloyloxyethyltrimethylamonium chloride (Q9).

This paper presents the systematic examination of the FRP of the cationic double-charged monomers 1,3-bis(*N,N,N*-trimethylammonium)-2-propylmethacrylate dichloride (di-M) and 1,3-bis(*N,N,N*-trimethylammonium)-2-propylacrylate dichloride (di-A) in aqueous solution within a broad range of monomer concentration and at different temperatures. The two positive charges of the double-charged monomer molecules are located at two different quaternary ammonium groups, which are structurally separated by the –CH_2_–CH–CH_2_– group. These monomers yield PEL with a theoretical/structural charge spacing of only ≈0.12 nm. Studies on the mono-charged analogue acryloyloxyethyltrimethylamonium chloride (Q9) have been included for comparison because appropriate reference data were missing. Analysis of the electrochemical properties and the solution behavior of the highly charged PEL molecules completed the study. The chemical structures of the three monomers are shown in Scheme 1.

The selection of these three monomers did not only allow for investigating extreme electrostatic influences on the polymerization by comparing mono- and double-charged monomers but also permitted the evaluation, at this stage at least qualitatively, of the impact of the hydrophobicity of the backbone, by comparing acrylic and methacrylic structures. Different kinetic mechanistic behavior of acrylic and methacrylic structures has been reported previously [19,20]. The structural difference adds more complexity to the polymerization studies.

After preliminary homopolymerization studies on di-M [21] and the study of its copolymerization with the aim to evaluate the reactivity of di-M in comparison to mono-charged monomers and acrylamide [22], this is, to our knowledge, the first more detailed study on such highly and permanently charged monomers.

## 2. Materials and Methods

### 2.1. Materials

1,3-bis(*N,N,N*-trimethylammonium)-2-propylmethacrylate dichloride and 1,3-bis(*N,N,N*- trimethylammonium)-2-propylacrylate dichloride were supplied by Taminco N.V. (Gent, Belgium) as powders [23]. Acryloyloxyethyltrimethylamonium chloride, 80 wt % in water, was purchased from Sigma-Aldrich GmbH (Schnelldorf, Germany). 2,2′-Azobis(2-methylpropionamidine) dihydrochloride (AMPHC) (Sigma-Aldrich, Steinheim, Germany) was employed as initiator. The water for all solutions and mixtures was bidistilled/deionized. A mixture of acetone/methanol, 75/25 vol %, HPLC-grade (Applichem-Axon Lab AG, Baden-Dättwil, Switzerland) was used to precipitate the polymers.

### 2.2. Polyelectrolyte Synthesis and Conversion Analysis

All polymer syntheses were performed in a 30 mL cylindrical glass reactor (*d* = 1.7 cm, *h* = 13 cm) equipped with stirrer, condenser, gas inlet, and a heating/cooling jacket. The reaction temperature was adjusted within ± 1 K. Oxygen was removed prior to polymerization by purging with argon for 40 min at room temperature (rt). A short induction period of up to 3 min was observed for the batch polymerizations and confirmed by conversion control. This was the time between addition of the initiator to the monomer solution at 293 K and increasing the temperature to the reaction temperature, typically 323 K. The onset of the polymerization (*t*_0_) was set at the time when the temperature in the reactor had reached 323 K. Samples of 0.3–0.4 g were withdrawn in defined time intervals. Thermal decomposition of the initiator was insignificant during the degassing process. Experiments were repeated at least twice to ensure accuracy and experimental reproducibility. The data reported in Section 4 are average values of at least two experimental repeats. Table 1 lists all polymerizations and conditions.

The equipment for the conversion analysis was described previously [21,22]. Briefly, the residual monomer was analyzed using an HPLC system composed of an injector 7725i Rheodyne (Sigma-Aldrich, Buchs, Switzerland), a stainless steel filter, a pre-column (Waters, Milford, MA, USA), a pump L-6000, and a UV detector L-4000H operating at 193 nm (Hitachi, Japan). The stationary phase was a Nova-Pak cartridge housed in a radial compression system, RMC 8 × 10 (Waters) operating at 180 kg/cm^2^. The mobile phase was a mixture of 50/50 wt % acetonitrile/bidistilled water containing 0.005 mol/L dibutylamine phosphate adjusted with *o*-phosphoric acid to pH = 3. The flow rate was set at 3.1 mL/min. The samples withdrawn from the reactor were first mixed with 3 mL of acetone/methanol to precipitate and isolate the polymer. 1 mL of the supernatant containing the monomer was centrifuged to complete the separation. Subsequently, 0.5 mL of the supernatant was diluted in 5 mL water to ensure monomer peak detection within the detectable range. Finally, 0.02 mL of this diluted solution was injected into the HPLC. The HPLC system was calibrated using monomer solutions of precise concentrations in the range 5 × 10^2^ to 5 × 10^4^ ppm covering the operating range of the detector. The peak area served as calibration parameter. The overall rate of polymerization, *R_P_,* was calculated from the slope −d[M]/d*t* at conversions where the linear regression was characterized by *r^2^* > 0.99. In Figure S1 (Supplementary Materials) it is shown how higher initial monomer concentration, higher *R*_P_, limits the linear range to shorter polymerization periods. The accuracy and reproducibility of the procedure was reported in [21], as reproducibility within ≈±1%.

### 2.3. Polyelectrolyte Characterization

The intrinsic viscosity, [η], at (293 ± 0.2) K was obtained from dilution series of the PEL in 0.5 mol/L NaCl (adjusted with HCl to pH 3.5) using a Viscologic TI 1 (Laser Instrument Diffusion, Nice, France) equipped with an Ubbelohde capillary of 0.58 mm diameter. The experimental data were analyzed according to Huggins and Schulz-Blaschke [24,25]. The same equipment was used to study the chemical stability of poly(di-A), poly(di-M), and poly(Q9) in aqueous solution at different pH. The flow time as a function of the storage time was monitored for this purpose.

An OSMOMAT 090 membrane osmometer equipped with cellulose acetate membranes (Gonotec GmbH, Berlin, Germany) served to determine the number average molar mass, *M_n_*, at (293 ± 0.2) K in 0.5 mol/L NaCl degassed solution.

A pH/Ion meter 692 with temperature compensation, equipped with a chloride ion selective electrode (Metrohm, Zofingen, Switzerland), was used to measure the counterion activity in diluted aqueous PEL solutions (chloride concentration: 5 × 10^−4^ to 3.5 × 10^−3^ mol/L) as described previously [26,27].

### 2.4. Analysis of Monomer, Monomer Solution, and Polymer Solution Properties

The dynamic viscosity of the aqueous monomer and polymer solutions was measured at (323 ± 0.2) K in a Lovis 2000 M/ME viscometer-densitometer (Anton Paar GmbH, Graz, Austria) using either a capillary of 1.59 mm (Mat. No. 93095) or 2.5 mm (Mat. No. 93097) diameter and rolling gold balls (Mat. No. 20659) at angles in the range of 20° to 70°. This combined instrument was also used for a part of density measurements.

The partial specific volume of the monomers was obtained from their dilution series in water according to

(1) $$\rho =\rho_{0}+\left(1-\rho_{0}\bar{\nu }\right)c$$

using a high-precision digital density measuring system DMA60/DMA602 (Anton Paar GmbH, Graz, Austria) at (293 ± 0.01) K. The parameters, $\rho_{0},\;\bar{\nu }$, and *c* denote the density of the solution, the density of the solvent, the partial specific volume of the solute, and the weight concentration of the solute.

For surface tension measurements by the Du Nouy ring method, a Sigma 703 Digital Tensiometer (KSV Instruments Ltd., Helsinki, Finland) was used.

## 3. Theoretical Background and Basic Equations

### 3.1. Free-Radical Polymerization Kinetics

FRP follows the overall rate equation

(2) $$R_{P}=k{\left[M\right]}^{\alpha }{\left[I\right]}^{\beta }$$

where α is the reaction order of the monomer concentration [M], β is the reaction order of the initiator concentration [I], and *k* is the overall polymerization rate constant, which obeys the Arrhenius equation

(3) $$k=A\cdot e^{-\frac{E_{a}}{RT}}$$

In Equation (3) *k, T, R, A,* and *E*_a_ are the rate constant, the Kelvin temperature, the universal gas constant, the pre-exponential factor (collision frequency factor), and the overall activation energy.

If no side reactions occur, the polymerization rate equation becomes

(4) $$R_{P}=-\frac{d\left[M\right]}{dt}=k\left[M\right]{\left[I\right]}^{0.5}=k_{p}{\left(\frac{k_{d}f}{k_{t}}\right)}^{0.5}\left[M\right]{\left[I\right]}^{0.5}$$

a special case of Equation (2) with α = 1 and β = 0.5, and where *k*_d_, *k*_p_, *k*_t_ are the rate coefficients of initiator decomposition, chain propagation and termination, and *f* is the initiator efficiency factor. The slope of the double-logarithmic plot of *R*_P_ *vs.* [I] or [M] directly yields α and β [28].

During the polymerization of di-M, di-A and Q9, radical transfer according to Equation (5), known as Mayo equation [29], cannot be excluded.

(5) $$\frac{1}{P_{n}}=\frac{k_{t}R_{P}}{k_{p}^{2}{\left[M\right]}^{2}}+C_{M}+C_{S}\frac{\left[S\right]}{\left[M\right]}+C_{I}\frac{\left[I\right]}{\left[M\right]}+\cdots$$

*P*_n_ is the number average degree of polymerization, and the coefficients *C*_M_, *C*_S_, and *C*_I_, which stay for the ratios of the rate coefficients of the transfer reaction (*k*_*trans*M_, *k*_*trans*S_, *k*_*trans*I_) to the rate coefficient of the propagation reaction (*k*_p_), specify the radical transfer to the monomer, solvent and initiator, respectively. Equation (5) neglects bimolecular termination by combination.

For polymerizations toward only low monomer conversion, radical transfer to the polymer can usually be neglected. The same holds for the radical transfer to the initiator because the initiator concentration is mostly much lower than the monomer concentration. A kinetic analysis presented previously confirmed the absence, or at least insignificance, of primary radical termination (PRT) and degradative chain transfer (DCT) to the initiator for the polymerization of di-M [21]. Then, Equation (5) can be rewritten as

(6) $$\frac{1}{P_{n}}-\frac{k_{t}R_{P}}{k_{p}^{2}{\left[M\right]}^{2}}=C_{M}+C_{S}\frac{\left[S\right]}{\left[M\right]}$$

where only the radical transfer to the monomer and solvent remains. Equation (6) will be used for the analysis of the initial stage of polymerization.

### 3.2. Autoacceleration

According to Equation (2), decrease of the reaction rate is expected during progressing chain polymerization, because both monomer and initiator concentration decrease simultaneously. However, the opposite behavior, increase of the reaction rate designated as autoacceleration, was observed for some polymerizations. In such cases, the conversion curves are subdivided into three stages: An initial stage, where the rate of polymerization is almost constant due to the negligible consumption of monomer and initiator; an intermediate stage, where the polymerization rate increases dramatically; and a final stage, where the rate approaches 0. The autoacceleration observed in the second stage is known as the so-called gel effect or Trommsdorff effect [30,31,32,33,34]. Noteworthy, in this context the term gel does not refer to the formation of crosslinked polymer molecules. The gel effect is a phenomenon observed under isothermal reaction conditions. It is of practical and scientific interest. Accelerative phenomena including chain entanglements, depletion of short chains, free and excluded volume, organized monomer systems, and system heterogeneity have been presented and discussed in detail by Odian [35] based on appropriate original references.

While the molecular mechanism of the gel effect is still debated and subject to active research [36], and no final commonly accepted explanation is available, its association with the decrease of the termination rate due to restriction of the chain mobility is widely accepted. The chain mobility decreases as the result of the higher viscosity associated with monomer to polymer conversion.

Increase of the viscosity due to polymer formation has been intensely considered and discussed. However, almost no attention has been given particularly to viscosity increase as the result of varying medium conditions such as change of the ionic strength due to counterion condensation. The latter could be expected for the polymerization of ionic monomers, which yield PEL of such high charge density that a substantial portion of previously free counterions condense.

### 3.3. Permanently Charged Polyelectrolytes in Solution

**Scheme 2:**
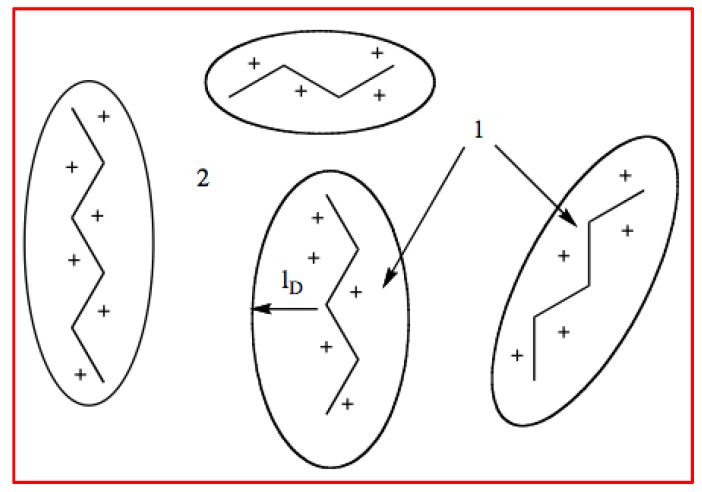
Polyelectrolyte (PEL) chains in solution. (**1**) PEL occupied region and extension of the ionic atmosphere determined by the Debye lengths, *l*_D_; (**2**) free volume region.

Ionic monomers behave in aqueous solution similarly to low molar mass salts. Upon dissociation in water, all their anions and cations contribute to the ionic strength, and the salt concentration is inversely proportional to the Debye length, *l*_D_. Equation (7) specifies *l*_D_ for monovalent salts, such as NaCl, which was used to vary the ionic strength in the polymerizing batches (Table 1). Different, counterion condensation occurs for PEL, which have charge distances less than the Bjerrum length, *l*_B_ in Equation (9), to an extent defined by the Manning charge-density parameter, ξ in Equation (10), and not all counterions and polymer backbone charges contribute to the ionic strength and Debye length. Hence, the total ionic strength in the polymerizing batch, where ionic monomer molecules become assembled into PEL chains, decreases while *l*_D_ according to Equation (8) increases. Increase of *l*_D_ is accompanied by greater persistence length, polymer chain expansion, greater hydrodynamic and excluded volume, and consequently, by reduction of the free volume as schematically shown in Scheme 2.

Equations (7) and (8) serve to show the general influences on *l*_D_ qualitatively. Overall, the Debye length is the main parameter, which determines the electrostatic contribution to the persistence length and to the excluded volume of PEL molecules in solution. Despite intense discussion, there is no scientific consensus on the accurate calculation of *l*_D_ for asymmetric electrolytes including PEL [37,38,39]. The majority of theoretical approaches and experimental proofs are restricted or apply to specific conditions, for example, limited concentration ranges, defined chain lengths, charge density, or chain flexibility. Further is debated whether the concentration or the activity is the more appropriate term [37]. A comprehensive theoretical presentation related to *l*_D_ including references would by far exceed the purpose of the present study. At the current state of experimental results, the discussion will be limited to qualitative evaluation of potential electrochemical influences on the polymerization behavior. Considering the monomers di-M and di-A as 2:1 electrolytes [40], values smaller than those calculated from Equation (7) will be obtained. This will also impact the use of Equation (8) if both added monovalent salt and double-charged monomer molecules are simultaneously present.

(7) lD=[8πNAlB(cs)]−12

(8) lD=[4πNAlB(ξ−1cp+2cs)]−12

with the Bjerrum length

(9) $$l_{B}=\frac{e^{2}}{4{\pi \varepsilon }_{0}\varepsilon_{r}k_{B}T}$$

and the Manning charge-density parameter [41], from which the fractions of free and condensed counterions can be calculated as 1/ξ and 1–(1/ξ), respectively.

(10) $$\xi =\frac{l_{B}}{b}$$

Here are *N*_A_ Avogadro’s number, *c*_p_ the PEL concentration, *c_s_* the monomer concentration or the sum of monomer and added salt concentration, *e* the electron charge, ε_0_ the dielectric permittivity of the vacuum, ε*_r_* the relative permittivity of the solvent, *k*_B_ the Boltzmann constant, *T* the Kelvin temperature, and *b* the structural charge spacing. With *l*_B_ ≈ 0.712 nm in water at 293.15 K and *b* ≈ 0.125 nm for poly(di-M) and poly(di-A), but *b* ≈ 0.25 nm for poly(Q9), it becomes ξ ≈ 5.7 for poly(di-M) and poly(di-A), and ξ ≈ 2.8 for poly(Q9). Supplementary Materials provide details on the determination of these values and basic parameters.

Differently than for kinetic-mechanistic FRP studies, where the double bond of the monomer molecule reacts and the monomer concentration is used as molar concentration (mol/L), charge-related concentrations have to be used for electrochemical studies and analyses. In the case of monomer molecules with only one charge, such as Q9 or DADMAC, mol/L can be used for expressing the salt concentration, the ionic strength, and to calculate the *Debye* length. Appropriately, the unit monomol/L has been used for the electrochemical PEL concentration, which refers to a single charge on one monomeric unit in the PEL backbone [26,27]. However, for double-charged monomers, such as di-M and di-A, both charges contribute to electrochemical parameters and have to be included in theoretical calculations and electrochemical data interpretation. Relying on these considerations, charge-related concentrations (mol_ion_/L), which are equal to the chloride concentration, were used in this paper for mono- and double-charged monomers and their PEL when electrochemical issues were concerned. For di-M and di-A, 1 mol/L = 2 mol_ion_/L is valid, while 1 mol/L = 1 mol_ion_/L is valid for Q9, DADMAC and other mono-charged monomer molecules.

## 4. Results

### 4.1. Monomer and Monomer Solution Properties

As a prerequisite for detailed polymerization studies, basic characteristics of the two new monomers di-M and di-A were analyzed. These included solubility, partial specific and partial molar volume, as well as the concentration-dependent viscosity, density and surface tension of the monomer solutions.

For di-M, the maximum obtainable concentration in water at rt was ≈1.9 mol/L (599 g/L), whereas di-A could be dissolved up to ≈2.2 mol/L (664 g/L).

Table 2 presents the partial specific and partial molar volumes as well as their comparison with model calculations [42,43]. The concentration dependence of the density of di-M, di-A, and Q9 aqueous solutions was linear up to high concentrations. (Figure S2, Supplementary Materials). According to Equation (1), the slope of ρ = *f*(*c*) plots yielded the partial specific volume and, by multiplication with the molar mass, the partial molar volume. Compared to theoretical calculations, slightly higher values were obtained experimentally.

**Figure 1:**
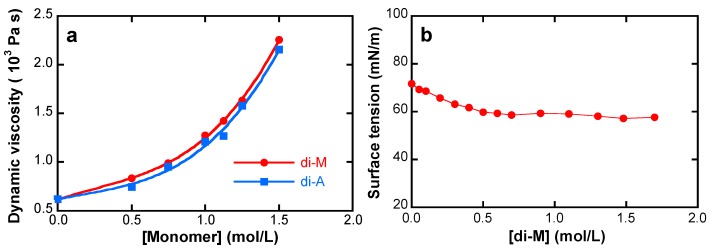
(**a**) Dynamic viscosity of aqueous di-M and di-A solutions at the polymerization temperature *T* = 323 K; (**b**) Surface tension of aqueous di-M solutions at *T* = 297 K, (accuracy ± 2 mN/m).

Figure 1 shows the influence of the monomer concentration on the dynamic viscosity and surface tension of aqueous monomer solutions in the concentration range, which was addressed by polymerization studies. The dynamic viscosity increases exponentially but remains relatively low. The surface tension reduces up to [di-M] ≈ 0.6 mol/L, but levels off toward higher [di-M].

### 4.2. Polymerization to High Conversion

**Figure 2:**
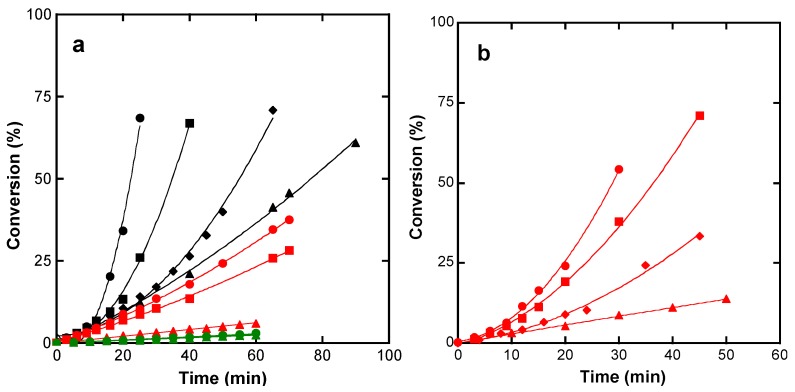

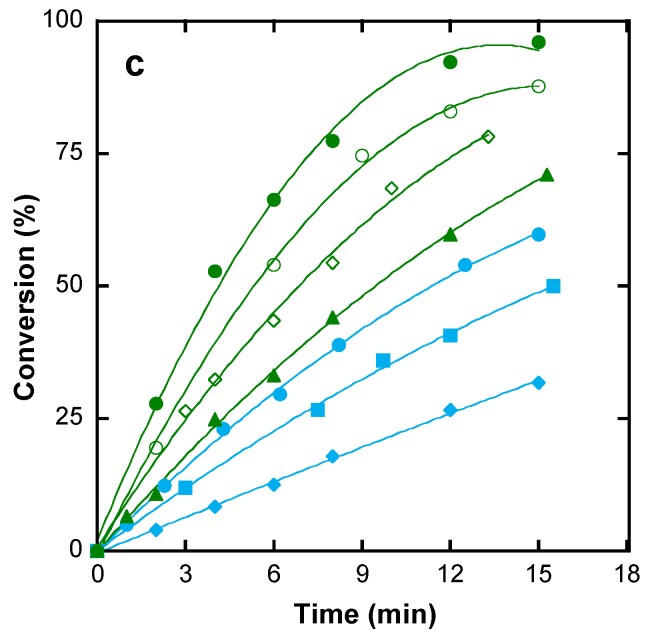
Conversion curves of free-radical polymerization (FRP) of di-M, di-A and Q9 at *T* = 323 K (all initial monomer and initiator concentrations in mol/L); (**a**) [di-M]_0_ = ▲0.3, ◆0.45, ●0.6, ▲0.8, ■1.25, ●1.35, ▲1.4, ◆1.45, ■1.5, ●1.6, [I] = 1.6 × 10^−2^; (**b**) [di-A]_0_ = ▲0.86, ◆0.98, ■1.23, ●1.35, [I] = 1.6 × 10^−2^; (**c**) [Q9]_0_ = ◆0.1, ■0.15, ●0.2, ▲0.3, ♢0.4, ◯0.5, ●0.6, [I] = 4.92 × 10^−3^. (Symbols and colors support the visualization of comparable concentrations and concentration ranges; blue < green < red < black; in 2a, the three lowest concentrations (green) overlay.)

Polymerizations of di-M, di-A and Q9 up to high conversions and over a wide range of the initial monomer concentration served to analyze the general polymerization behavior (Figure 2).

For both double-charged monomers, increase of the slope with polymerization time is visible in Figure 2a,b for higher initial monomer concentrations, reflecting the increase of *R_P_*. Contrary, the polymerization rate of Q9 follows the behavior expected for free radical FRP (Figure 2c), the slope of the conversion curves is constant or decreases with time due to reduction of the monomer and initiator concentrations.

Comparing the same concentrations of di-M and di-A, for example 1.35 mol/L, the polymerization of di-A proceeds faster than the polymerization of di-M. At 30 min, the conversion is 13.5% for di-M but 54.2% for di-A (Figure 2a,b). The Q9 polymerization proceeds much faster than the di-M and di-A polymerizations, even though a lower initiator concentration was used. For example, at [di-M]_0_ and [Q9]_0_ = 0.3 mol/L and *t* = 15 min, the conversion of di-M is only 0.6% but 70% for Q9 (Figure 2a,c).

To identify the reason for the increasing polymerization rate, the molar mass of di-M polymers was analyzed and presented in Table 3. For two different initial di-M concentrations, the data in Table 3 confirms higher molar masses at higher conversion.

The number average molar mass, *M*_n_*,* and the degree of polymerization, *P*_n_, of poly(di-M) samples were calculated from intrinsic viscosity data according to Equation (11). Details and validity range of this relationship are presented in Section 4.4.1.

(11) $$\left[\eta \right]=8.36\times 10^{-4}{M_{n}}^{0.96}\;\left[\text{mL}/g\right]$$

**Figure 3:**
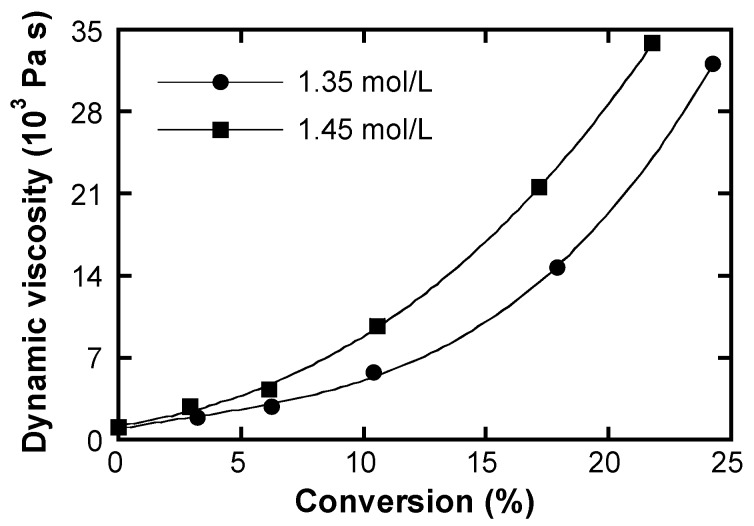
Increase of the dynamic viscosity during the free radical polymerization of di-M; initial monomer concentrations: [di-M]_0_ = 1.35 mol/L and 1.45 mol/L, [I] = 1.6 × 10^−2^ mol/L, *T* = 293 K.

Already the initial polymerization periods, up to 24.3% conversion for [di-M]_0_ = 1.35 mol/L and 21.8% conversion for [di-M]_0_ = 1.45 mol/L, are accompanied by remarkable exponential increase of the dynamic viscosity in the polymerizing batches (Figure 3).

To identify and demonstrate the presence and importance of electrostatic influence, low molar mass electrolyte (NaCl) was added to the monomer solution to achieve higher ionic strength in the di-M batch. Higher ionic strength was expected to reduce the viscosity and to this end the acceleration effect. Table 4 presents the results.

**Figure 4:**
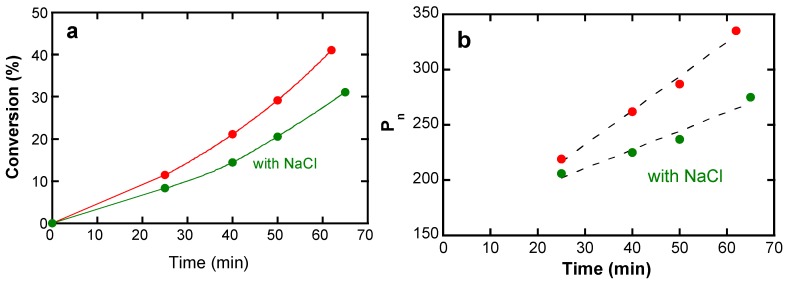

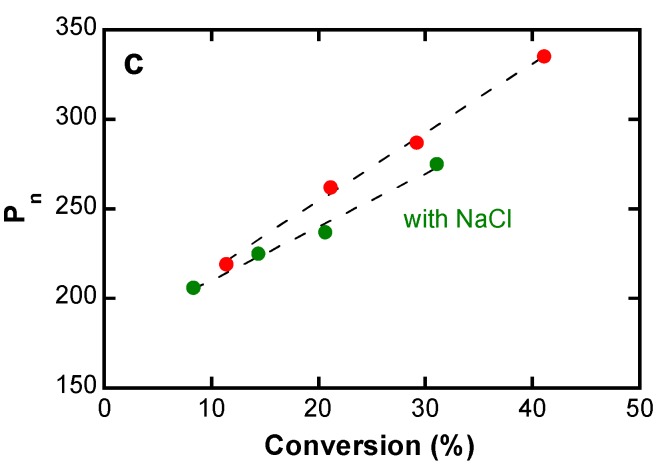
Polymerization of [di-M]_0_ = 1.35 mol/L without and with addition of [NaCl] = 1 mol/L, [I] = 1.6 × 10^−2^ mol/L, *T* = 323 K; (**a**) Conversion *vs.* time; (**b**) Degree of polymerization, *P*_n_, *vs.* time; (**c**) *P_n_ vs.* conversion.

Figure 4 illustrates the comparison of di-M polymerizations without and with addition of NaCl. Both conversion and *P*_n_ reduced upon addition of low molar mass salt.

### 4.3. Polymerization Kinetics

After having presented data on the polymerization behavior of di-M, di-A, and Q9 up to high conversion, the focus will now be on the results of kinetic studies limited to the range of low monomer conversion.

#### 4.3.1. Effect of the Monomer Concentration

**Figure 5:**
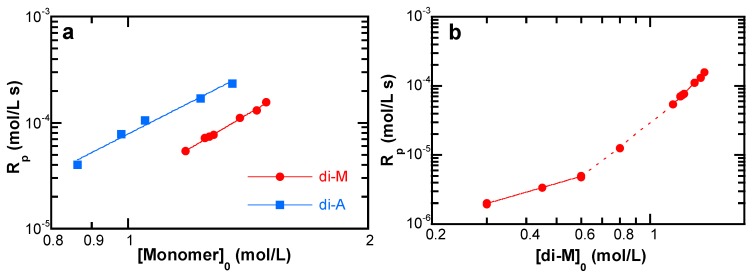

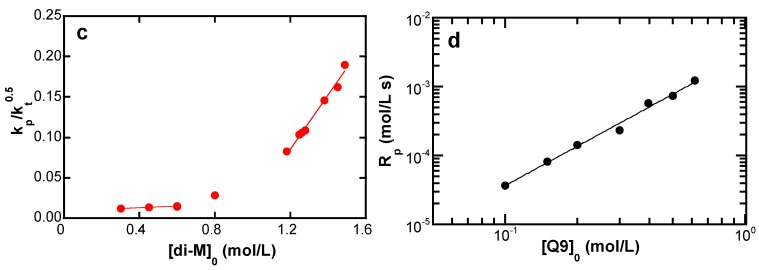
(**a**) Determination of α for [di-M]_0_ ≥ 1.26 mol/L and [di-A]_0_ ≥ 0.8 mol/L; (**b**) *R*_P_ for 0.3 < [di-M]_0_ < 1.6 mol/L; (**c**) *k*_p_*/k*_t_^0.5^ for 0.3 < [di-M]_0_ < 1.6 mol/L; (**d**) α for 0.1 ≤ [Q9]_0_ ≤ 0.6 mol/L; (**a**)–(**c**) [I] = 1.6 × 10^−2^ mol/L; (**d**) [I] = 4.92 × 10^−3^ mol/L; *T* = 323 K.

The strong and non-ideal influence of the initial di-M concentration on the polymerization rate, which according to Equation (2) lead to α = 4.40 ± 0.16 for a confidence interval of 95% [21], was confirmed by more experiments (Series 5, Table 1). In addition, α = 3.50 ± 0.10 (Series 6, Table 1) was calculated for di-A for the same confidence interval (Figure 5a).

In Figure 5a, the monomer concentration is in the range [di-M]_0_ ≥ 1.26 mol/L and [di-A]_0_ ≥ 0.8 mol/L. Formally, monomer concentration-independent monomer exponents were calculated. The behavior changed at lower monomer concentrations (Figure 5b). The slope decreased with [di-M]_0_ yielding α = 1.3 ± 0.05 for [di-M]_0_ < 0.6 mol/L. A transition range was observed for 0.6 < [di-M]_0_ < 1.2 mol/L. Figure 5c shows the same trend for *k_p_/k_t_*^0.5^ *vs.* [di-M]_0_. For the analysis of the dependency *k_p_/k_t_*^0.5^ = *f* ([di-M]_0_) it was assumed that *k_d_* is not monomer concentration-dependent and the initiator efficiency factor *f* is constant. These assumptions are justified relying on studies for a number of systems at low conversion and at relatively wide ranges of monomer concentration [28]. *k_d_* was taken as 1.9 × 10^−5^ s^−1^ [44] and *f* was set as 1 [45].

The much higher reactivity of Q9 did not allow experiments at monomer concentrations comparable to [di-M]_0_ and [di-A]_0_. Therefore, [Q9]_0_ < 0.6 mol/L was chosen. From the plot in Figure 5d, α = 1.9 ± 0.02 was calculated.

#### 4.3.2. Effect of the Temperature

**Figure 6:**
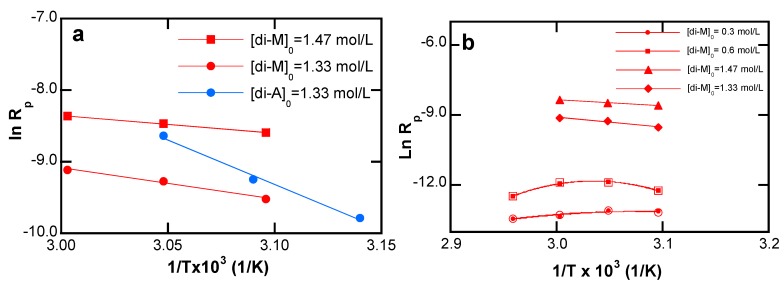
Arrhenius-type plots for (**a**) di-M and di-A polymerizations at 318 < *T* < 333 K; (**b**) di-M polymerizations at 323 < *T* < 338 K; [I] = 1.6 × 10^−2^ mol/L (empty and filled markers refer to the results of two independent polymerization series).

Temperature effects were analyzed for [di-M]_0_ ≥ 1.33, 0.6, and 0.3 mol/L, as well as [di-A]_0_ = 1.33 mol/L. From polymerizations at 318 K < *T* < 338 K (Series 8 and 9, Table 1), *E*_a_ and *A* were calculated according to Equation (3) (Figure 6a,b, Table 5).

For [di-M]_0_ ≥ 1.33 mol/L and [di-A]_0_ = 1.33 mol/L, ln *R_P_* increased with *T*, and *E*_a_ could be calculated as usual. However, at low [di-M]_0_, 0.3 and 0.6 mol/L, unusual behavior was observed, ln *R*_P_ first increased with *T*, passed a maximum, before declining at higher temperature.

#### 4.3.3. Side Reactions

**Figure 7:**
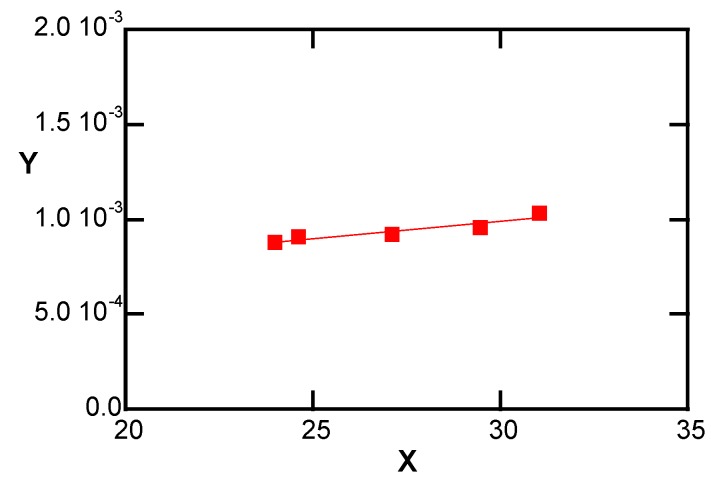
Radical transfer to the monomer di-M and the solvent water at *T* = 323 K, [I] = 1.65 × 10^−2^ mol/L (average values of two independent polymerization series).

Experimental data of di-M polymerizations performed at constant initiator concentration and five monomer concentrations in the range of 1.2 ≤ [di-M]_0_ ≤ 1.5 mol/L, and Equation (6) were used to analyze radical transfer to monomer and solvent as potential side reactions. Figure 7 shows the plot $Y=\frac{1}{P_{n}}-\frac{k_{t}R_{p}}{k_{p}^{2}{\left[M\right]}^{2}}$ *vs.* $X=\frac{\left[S\right]}{\left[M\right]}$.

Duplicated experiments yielded *C*_M_ = 4.31 × 10^−4^ and *C*_S_ = 1.87 × 10^−5^ (*R*^2^ = 0.881) for the radical transfer coefficients to di-M and water, respectively.

### 4.4. Polyelectrolyte and Polyelectrolyte Solution Properties

#### 4.4.1. Intrinsic Viscosity and Molar Mass

Addition of at least 0.5 mol/L NaCl was necessary to obtain linear Huggins [24] and Schulz-Blaschke [25] plots by dilution viscometry. Table 6 shows the intrinsic viscosity of the samples used for the determination of *M_n_* by membrane osmometry.

These [η]/*M*_n_ pairs served to derive the [η]–*M_n_* relationship presented as Equation (11) in Section 4.2 for poly(di-M) in the molar mass range 8 × 10^4^ < *M*_n_ < 2 × 10^5^ g/mol. (Figure S3, Supplementary Materials). The last two lines in Table 6 refer to polymers of high intrinsic viscosity obtainable from polymerizations at the solubility limits of di-M and di-A, 1.9 and 2.2 mol/L, respectively. Due to autoacceleration effects at these high concentrations, much higher molar masses were produced.

It is worth mentioning that the hydrodynamic volume of the PEL in water exceeded the hydrodynamic volume in 0.5 mol/L NaCl by a factor of 5–8. For example, for poly(di-M) of [η] = 58 mL/g in 0.5 mol/L NaCl, the reduced viscosity, η_red_, in water increased to 460 mL/g at *c* = 7.2 × 10^−4^ g/mL, *T* = 293 K.

#### 4.4.2. Counterion Condensation

Counterion activity coefficients, *f*_a_, as low as 0.11 for poly(di-A) and 0.12 for poly(di-M) obtained from counterion activity measurements by direct potentiometry according to *a = f*_a_ × *c* [26] (*a*: counterion activity, *c*: total chloride ion concentration) confirmed the theoretically expected high level of counterion condensation. There is good agreement with theoretical values *f*_a_ = 0.102 calculated according to Manning [46,47] and *f*_a_ = 0.118 according to Gueron [48]. Figure S4 (Supplementary Materials) shows the experimental results.

#### 4.4.3. Comparison of Hydrolytic Stability

**Figure 8:**
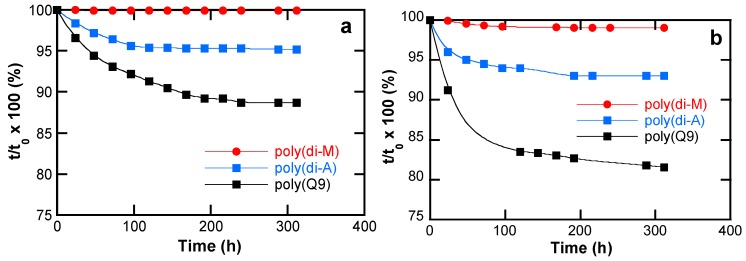
Hydrolytic stability of poly(di-M), poly(di-A), and poly(Q9) in water at (**a**) pH = 7.5; (**b**) pH = 9; *t/t*_0_ × 100 (percentage of *t*_0_) represents the reduction of the viscosity; concentrations and initial flow times: poly(di-M) = 12 g/L (*t*_0_ = 598 s), poly(di-A) = 12 g/L (*t*_0_ = 324 s), poly(Q9) = 0.54 g/L (*t*_0_ = 376 s); *T* = 293 K.

Exposure of poly(di-M), poly(di-A), and poly(Q9) to basic solution conditions, pH 7.5 and 9, revealed different hydrolytic stability. The flow time of the PEL solution through a viscometer capillary, as a measure of the solution viscosity, was controlled over a period of two weeks. Normalization to the flow time at *t_0_* is shown in Figure 8. The methacrylic PEL poly(di-M) resists hydrolysis at pH = 7.5 and pH = 9, whereas the acrylic PEL poly(di-A) and poly(Q9) do not. The viscosity drop was most pronounced at pH = 9, with poly(Q9) being the most instable polycation.

## 5. Discussion

The polymerization of ionic monomers yields PEL. For the case ξ >1 (Equation (10)), counterion condensation occurs, *i.e.*, the ionic strength in the polymerizing batch decreases upon assembling the monomer molecules to polymer chains. The higher the structural charge density of the produced PEL, the more counterions condense. The subject of the present study was the polymerization of the two double-charged monomers di-M and di-A (Scheme 1).

**Scheme 3:**
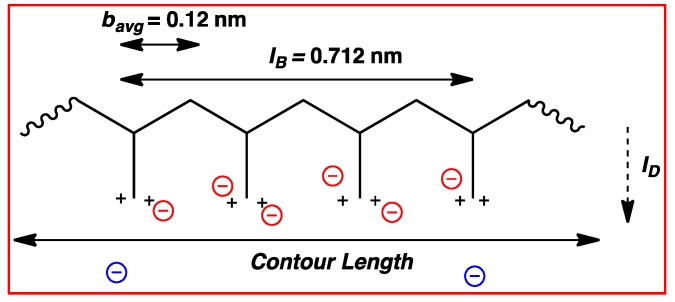
Illustration of counterion condensation for PEL of double-charged cationic vinyl monomers; (condensed, free); *b_avg_*: average theoretical/structural charge spacing; *l*_B_: Bjerrum length; *l*_D_: Debye length.

Di-M and di-A yield cationic PEL with ξ ≈ 5.7. For this high Manning charge-density parameter, the fraction of condensed counterions is predicted as 1–(1/ξ) = 0.82 [41], *i.e.*, 82% of the chloride counterions are necessary to enhance the theoretical/structural charge spacing (0.12 nm) to the Bjerrum length (0.712 nm) (Scheme 3). In the following, the experimental results presented in Section 4 are analysed and discussed in detail.

### 5.1. Monomer and Monomer Solution Properties

The monomers di-M and di-A (Scheme 1) were well soluble in water. The 15% higher molar solubility of di-A may be attributed to the 5% lower molar mass of di-A compared to di-M and to the absence of the hydrophobic methyl group in the neighborhood of the reactive double bond. Inspecting the concentration dependence of the density (Figure S2) and dynamic viscosity (Figure 1a), both monomers behave as expected for low molar mass salts [49], although, the exponential increase of the dynamic viscosity is more pronounced than for inorganic low molar mass salts. Contrary, the surface tension of the aqueous di-M solution decreases from 71.6 ± 0.2 mN/m (water value) to 59.3 ± 0.2 mN/m at [di-M] = 0.6 mol/L before leveling off to 57.6 ± 0.2 mN/m at [di-M] = 1.6 mol/L (Figure 1b). Such behavior is typical for surfactants, while the surface tension of aqueous low molar mass salt solutions generally increases continuously toward high salt concentrations [50].

**Figure 9:**
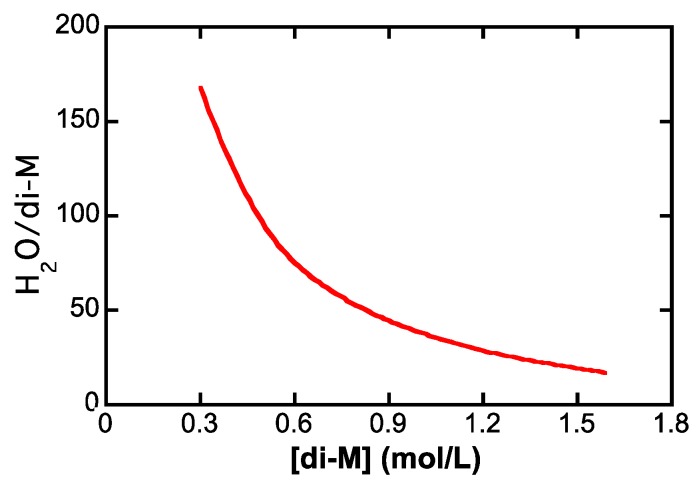
Number of water molecules available for the dissolution of one di-M molecule at different di-M concentrations.

**Scheme 4:**
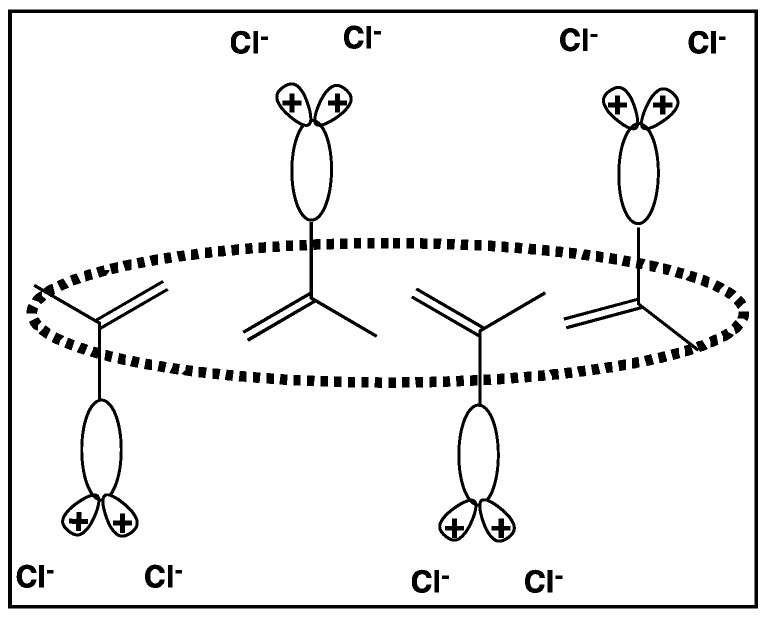
Association of di-M molecules due to the lack of water molecules in the range of high monomer concentrations.

Despite optically clear solutions at all monomer concentrations, the formation of organized/associated structures of the monomer molecules above a certain monomer concentration is probable (Scheme 4) due to lack of water molecules. The number of water molecules available per di-M molecule is presented in Figure 9 and supports this hypothesis. While at [di-M] = 0.3 mol/L, 168 water molecules are available to dissolve one di-M molecule, only 17 water molecules remain at [di-M] = 1.6 mol/L. This low number of solvent molecules could be insufficient for mono-molecular dissolution of the double-charged monomer cation and the two chloride counterions. Different reaction kinetics was reported for the polymerization of associated and isolated monomer molecules [14,51,52,53,54,55,56], and can therefore be hypothesized for the polymerization of di-M and di-A at the limits of high and low concentrations.

### 5.2. Autoacceleration

Autoacceleration was observed for the FRP of di-M and di-A in aqueous solution (Figure 2a,b). The following processes are expected to force the occurrence of autoacceleration:

- increase of the viscosity because of polymer chain formation,
- monomer association due to the lack of solvent for mono-molecular dissolution of the monomer molecules,
- enhancement of the viscosity as the consequence of reduced ionic strength due to counterion condensation upon monomer to polymer transformation.

While polymer chain formation and monomer association can occur for any monomer, counterion condensation is specific for ionic monomers which form PEL with structural charge spacing less than the Bjerrum length (Equation (9)). The total ionic strength in the polymerizing batches of di-M and di-A decreased strongly as quantified in Figure S5. Thereby, *l*_D_ can be expected to increase. Higher *l*_D_ will correspond to PEL chain expansion, higher persistence length and hydrodynamic volume, and will be accompanied by reduced free volume as shown in Scheme 2. To this end, the solution viscosity will depend not only on the concentration and the molar mass of the produced polymer but also on the ionic strength of the medium. If the PEL concentration rises relative to the monomer concentration, the electrostatic free volume will decline simultaneously with the ionic strength and will contribute to the enhancement of the dynamic viscosity in the batch as presented in Figure 3 for two di-M concentrations. Due to the current lack of reliable theoretical approaches and models for polyelectrolytes in concentrated aqueous solutions, the significance of electrostatic effects to induce autoacceleration can be neither confirmed nor excluded.

Another factor is the coil deformability (related with the persistence length). The electrostatic free volume and the coil deformability both affect the motion of the chains relative to each other [57]. Higher viscosity preferably reduces the motion of the larger PEL molecules. Assuming bimolecular termination, the overall rate of radical loss depends on the mobility of the PEL chains. Reducing the PEL chain mobility, termination becomes hindered relative to chain propagation, and autoacceleration is favored [58]. This was confirmed experimentally by higher molar mass at higher conversion. Table 3 presents the proof for two di-M concentrations. As expected, at similar conversions, the molar mass was higher for the higher initial monomer concentration.

Adding low molar mass electrolyte such as NaCl to the polymerizing batch increased the ionic strength, *l*_D_ and the volume occupied by the PEL decreased. This should lead to more free volume, lower viscosity, and less favored autoacceleration not least due to less hindered bimolecular termination reactions at lower viscosity. The comparably lower molar mass/degree of polymerization and less pronounced autoacceleration upon addition of salt are confirmed by the data in Table 4 and Figure 4. Figure 4c serves to illustrate that the differences of *P*_n_ increased with increasing conversion. Despite the already high di-M concentration (1.35 mol/L), the addition of 1 mol/L NaCl influenced the polymerization kinetics as expected from the electrochemical point of view. There is some evidence that polymer conformation changes occur in response to changes of the ionic strength, even at generally high ionic strength. The hydrodynamic volume expressed by the intrinsic viscosity increased by 5%–10% when using as solvent 0.5 mol/L NaCl instead of 1 mol/L NaCl.

Autoacceleration during the FRP of other cationic monomers than di-M and di-A was not reported for similar conditions [2]. Despite higher *R_P_* of the mono-charged Q9 as well as higher conversions and molar masses than achieved for di-M and di-A, Q9 polymerizations follow usual conversion curves with obviously no autoacceleration (Figure 2c). One could argue here that the Q9 concentrations were comparably low. The much higher reactivity of Q9 [22] prevented polymerizations at the same concentrations as used for the double-charged monomers. However, unpublished results previously obtained for the FRP of the less reactive DADMAC [22] also did not reveal any increase of *R_P_* with time (Figure S6), even not for initial DADMAC concentrations of 3 and 4 mol/L [2,59]. Hence, change of the electrochemical conditions in the polymerizing batch can be hypothesized as one reason for the autoacceleration phenomena observed when producing poly(di-M) and poly(di-A) with 82% counterions condensed. For poly(Q9) and poly(DADMAC) the percentage of condensed counterions is only 64% and 30%, respectively. Nevertheless, sufficiently high initial monomer concentrations of di-M and di-A were indispensable to induce autoacceleration. The individual contributions of monomer association, chain mobility, counterion condensation, steric effects, or specific feature of the monomers to the occurrence of the observed autoaccelerations currently cannot be quantified. While there is significant progress in predicting the static properties of PEL in solution, their dynamics is far from being completely understood [39]. This holds in particular for higher concentrations.

### 5.3. Polymerization Kinetics

The discussion of the polymerization kinetics includes concentration effects, temperature effects, side reactions, as well as electrostatic phenomena. The experimental basis are data obtained for the initial phase of the polymerization, where the slope −d[M]/d*t* was sufficiently linear (Figure S1) [11]. The following assumptions are justified at low conversion: negligible reduction of the initiator and monomer concentration, negligible change of the ionic strength, and no significant autoacceleration. The experimental results shown in Figure 5 and Figure 6 revealed non-ideal polymerization behavior even at low conversion. Importantly, different initial di-M and di-A concentrations constitute media of both different ionic strength and different quality of monomer dissolution.

#### 5.3.1. Reaction Order of the Initiator Concentration

The reaction order of the initiator of the di-M polymerization was analyzed and discussed previously [21]. Briefly, β obtained for di-M as 0.59 ± 0.03 (95% confidence) was considered as statistically different from the ideal value of β = 0.5 but independent of the di-M concentration in the investigated range. Analyzing the data according to Deb and Meyerhoff [60,61] as well as Ghosh, Mitra and Shukla [62,63] lead to the conclusion that neither PRT nor DCT took place, or were significant. Contrary to the predicted negative slopes for PRT and DCT, positive slopes were obtained plotting log(*R*_P_*^2^*/[I][M]^2^) = *f*(*R*_P_/[M]^2^) and log(*R*_P_*^2^*/[I][M]^2^) = *f*([I]/[M]), respectively [21]. Therefore, radical transfer to the monomer remained to explain the elevated initiator exponent. Previously reported transfer to methacrylic monomers supports this conclusion [64].

Radical transfer to the monomer di-M was confirmed by the experimental data shown in Figure 7. The positive slope, though small, refers to transfer to the solvent water, but could also be interpreted as different electrostatic interaction at different ratios water/di-M. Overall, at this stage it cannot be concluded free of doubt as to what extent radical transfer to di-M takes place and whether this is the only or main reason for the elevated initiator reaction order.

Radical transfer to di-A was not addressed in the present study. The occurrence of mid-chain radicals, which was reported for the FRP of acrylic but not methacrylic monomers, would have led to additional complications of the system.

#### 5.3.2. Monomer Concentration

The monomer exponent α was calculated for di-M, di-A, and Q9 at high monomer concentrations (Figure 5a,d), and for di-M at low monomer concentrations (Figure 5b). While Figure 5a,d suggest constant α values for higher concentrations of [di-M]_0_, [di-A]_0_, and [Q9]_0_, Figure 5b presents discontinuity in the *R*_P_ = *f*([di-M]_0_) plot upon extending the [di-M]_0_ range to lower concentrations. The slope decreased from 4.4 to 1.3, if [di-M]_0_, *i.e.*, the initial ionic strength, in the batches was lower. The unusually high monomer exponent of di-M and the plot in Figure 5c suggest that *k_p_* or *k_t_*, or both, varied with the initial monomer concentration.

Several authors discussed the polymerization behavior of DADMAC, for which a monomer exponent of 2 or 2.9 was reported, but partly controversially [65,66,67]. They considered viscosity effects, monomer association ,and change of the ionic strength differently. Topchiev *et al.* proposed *k*_t_ to decrease with increasing density and viscosity of the polymerization medium when the initial monomer concentration increased [65]. Others hypothesized the formation of monomer hydrophobic associates as well as reduced repulsive electrostatic forces between the positively charged growing radical and the cationic monomer molecules to cause an increase of *k*_p_ [66,67].

The lack of water molecules above a certain initial monomer concentration, estimated as about 0.6 mol/L for di-M from surface tension and viscosity data, could be assumed to interrupt the continuous solvent phase. Solubility could only be achieved upon associate formation, which results in reduction of the contact surface of the di-M molecules.

#### 5.3.3. Monomer Constitution

The higher reactivity of di-A in comparison to di-M is in agreement with differences reported for other acrylic and methacrylic monomers [20,68].

The different monomer exponents of di-A and di-M could result from different ability to self-organize. It is know that molecular arrangement depends on a number of competing forces, such as the relative magnitude of the attractive hydrophobic forces, the repulsive electrostatic forces between the charged groups, and the charged group hydration effect. Besides, the nature of the α-substituent of a vinyl monomer plays an important role in the kinetics of FRP [69,70]. Particularly the more hydrophobic character conferred by the α-methyl group of di-M seems to be responsible for the stronger organization of di-M monomers in aqueous solution. The analysis of the *Arrhenius* parameters (Table 5) supports this suggestion. Favorable arrangement by association being responsible for low values of the activation energy and the pre-exponential factor was reported and discussed by several authors [14,51,52,53,54,55,56]. Considering their arguments, a more favorable arrangement due to concentration-dependent association seems to be responsible for the low values of *E*_a_ and *A* estimated for di-M, Table 5. However, it is also important to recall that association enhances *R*_P_ because solvent molecules do not screen the reactive centers. Nevertheless, higher tendency to self-organization should not a priori be related to higher *R*_P_ when studying different monomer structures. Steric hindrance is another important factor, which has to be taken into account. Finally, the discovery that acrylate monomers can cause kinetic complications by the presence of mid-chain radicals, which largely influence the propagation and termination mechanism, should be considered when comparing di-A and di-M [71,72].

**Scheme 5:**
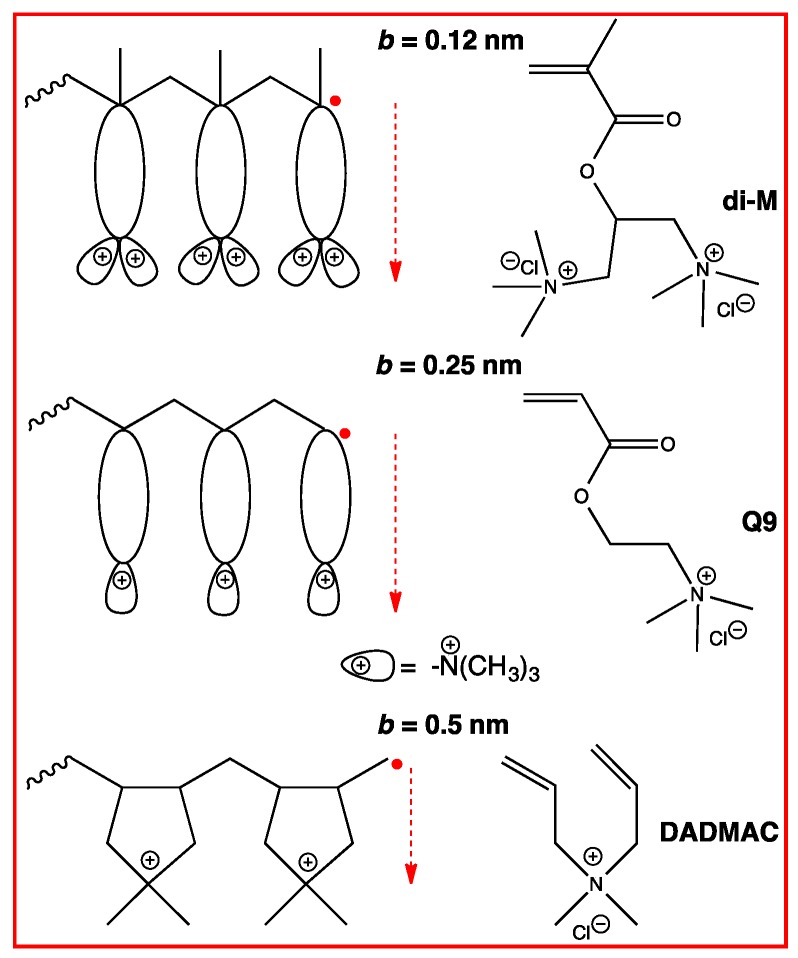
Monomer constitution and electrochemical parameters of di-M, Q9 and DADMAC; *b*: theoretical/structural charge spacing; • chain end radical position; red arrow: distance between radical position and cationic charge. (The free chloride counterions at the PEL chain end are not shown.)

The comparison of three constitutionally different types of cationic monomers (Scheme 5), which yield PEL with ξ >1 but different charge spacing, 0.5, 0.25, 0.12 nm, serves to highlight the importance and potential impact of Coulomb interaction on the FRP kinetics.

Despite the fact that counterion condensation reduces the theoretical/structural charge spacing, *b*, to the Bjerrum length (Equation (9)), independent of the charge of the monomer, differences exist at the radical chain ends. Theoretical studies have concluded inhomogeneity of counterion condensation on ionic oligomers and on the end of long PEL chains. The inhomogeneity is restricted to the length of the Debye length (Equation (8)) [73]. Relying on this, the electrostatic repulsion between the radical chain ends and their monomers should be in the order of di-A/di-M > Q9 > DADMAC, leading to expect the highest reactivity for DADMAC, what is not the case. Instead, the reactivity is Q9 > di-A/di-M > DADMAC. On the other hand, the distance between the radical position and the cationic charge is lowest for DADMAC. The latter may cause stronger repulsion.

Overall, only the direct and reliable determination of *k*_p_ and *k*_t_ for a wide range of experimental condition, as proposed, for example, by Lacík, Beuermann, or Buback *et al.* [74,75,76,77,78,79] will contribute to more insight into the polymerization mechanism in the presence of strong electrostatic interactions. There seems to be evidence that *k*_p_ behaves different for neutral/non-ionized and charged/fully ionized monomers. While *k*_p_ exponentially decreased toward higher concentration of non-ionized methacrylic acid, slight increase of *k*_p_ was found upon full ionization [14].

As a prerequisite for the reliable use of such sophisticated and powerful techniques and methods, potential complications arising from the constitution of di-A and di-M have to be evaluated in advance. This concerns in particular suitable stationary phases for SEC, which avoid electrostatic interaction with the highly charged PEL, the presence of the quaternary nitrogen as potential source for EPR complications [80], or variable ionic strength upon varying the monomer concentration.

The minimization of experimental complications was the reason for using the experimental conditions and conversion analysis described in Section 2.2. These were not sensitive to changes of the partial specific volume of the PEL at different ionic strength, or to variation of the medium during polymerizations.

#### 5.3.4. Temperature Effects

Ideally, the overall polymerization rate increases with increasing temperature. Such temperature influence was confirmed for high di-M and di-A concentrations, as shown by the Arrhenius plots in Figure 6a. These plots yielded for [di-A]_0_ = 1.33 mol/L an overall activation energy *E*_a_ = 103 kJ/mol (Table 5) in the range expected for the FRP of vinyl monomers,. Different, the value of *E*_a_ for the same di-M concentration was about three times lower and about five times lower for [di-M]_0_ = 1.47 mol/L. Such temperature-dependent change of *E*_a_ is in agreement with concentration-dependent monomer associate formation. However, steric hindrance by the methyl group lowers *R*_P_ compared to di-A.

For [di-M]_0_ ≤0.6 mol/L, nonlinear Arrhenius plots were obtained, unsuitable for estimating *E_a_*. Differently than for high di-M concentrations, ln *R*_P_ passes a maximum before decreasing upon temperature enhancement, suggesting a ceiling temperature, *T*_c_, at which the rates of propagation and depropagation are equal. At this dynamic equilibrium, *R*_P_ becomes zero. Using a procedure for determining *T*_c_ proposed by Yamada *et al.* [81], *T*_c_ = 339 K was calculated. Similar low *T*_c_ was reported for α-methyl styrene.

Strong electrostatic repulsion not only between the growing PEL radical and the double-charged monomer but also along the PEL chain could be an important factor supporting depropagation especially at low monomer concentration/low ionic strength. Incomplete counterion condensation is expected for oligomers and short PEL chains [73], as produced at low di-M concentration. Accordingly, the effective charge spacing is expected to become less than *l*_B_ and reinforces electrostatic repulsion, additionally favored by the more extended Debye length at low ionic strength.

### 5.4. Polyelectrolyte and Polyelectrolyte Solution Properties

#### 5.4.1. Intrinsic Viscosity and Molar Mass

The exponent 0.96 of the intrinsic viscosity-molar mass relationship established for poly(di-M) (Equation (11)) is slightly higher than found for other synthetic cationic PEL at comparable salt concentration [27] (p. 30). For comparison, exponents of 0.82, 0.71, 0.67, and 0.79 were reported for poly(DADMAC), poly(2-trimethylammoniummethylmethacrylate chloride), poly(4-vinylbenzyl- trimethylammonium chloride), and poly(allylammonium chloride), respectively. Such high exponents suggest chain stiffness and significant excluded volume effects, most pronounced for poly(di-M). This may result from Coulomb interactions, but also the size/volume of the pending side groups bearing two quaternary ammonium groups has to be considered.

Poly(di-M) samples of moderate molar masses were produced under polymerization conditions used for kinetic studies (Table 3, Table 4 and Table 6). Molar masses up to 4.8 × 10^5^ g/mol, *P*_n_ approx. 1500, were achieved upon polymerizing di-M and di-A at their highest possible concentrations (Table 6). The relatively wide range of molar masses could be interesting for practical applications of the PEL.

#### 5.4.2. Counterion Condensation

The experimentally determined counterion activity coefficients *f*_a_ = 0.11 for poly(di-A) and *f*_a_ = 0.12 for poly(di-M) (Figure S4) are much lower than the values so far reported for other synthetic PEL and in good agreement with values predicted by theoretical approaches [41,46,47,48]. The slightly higher value of poly(di-M) could be attributed to the steric influence of the methyl group at the backbone. The minimal increase of *f*_a_ at lower PEL concentrations could result from the contribution of a relatively low molar mass fraction or from experimental limitations [26]. The poly(di-M) samples used here have been dialyzed to remove all residual monomer but no fractionation was performed. Hence, end group effects cannot be excluded. The structural charge spacing, 0.12 nm, of poly(di-M) and poly(di-A) is in the range of the structural charge spacing of DNA, 0.17 nm.

#### 5.4.3. Hydrolytic Stability

The majority of industrial applications of PEL occur in aqueous solution. Some applications require high chemical stability while for others, degradation and decomposition could be important. Hence, knowledge on the chemical stability including the resistance to hydrolysis is crucial. Hydrolysis of ester bonds can principally occur at both acidic and basic conditions. However, primary esters such as present in poly(di-M) and poly(di-A), are often better hydrolyzed in basic environment [82]. Hydrolysis of poly(di-M) and poly(di-A) yields poly(methacrylic acid) and poly(acrylic acid), respectively. Both are non-permanently charged PEL with only one charge per monomeric unit. In addition, low molar mass ammonium salt is released. Upon hydrolysis, the viscosity of the solution will decrease and finally level off due to the reduced charge density at the polymer backbone and charge screening by the low molar mass ammonium salt. Moreover, the polycations will be turned into polyanions.

Different hydrolytic stability was assessed upon exposure of poly(di-M), poly(di-A), and poly(Q9) to basic conditions at pH 7.5 and pH 9 (Figure 8). Obviously, the methyl group confers good hydrolytic stability on poly(di-M), even at pH 9. Poly(di-A) and its mono-charged analogue were less stable. Whether there is any concentration or molar mass dependence of the hydrolysis, needs further detailed studies. The design of such studies will depend on the intended applications. The differences of the hydrolytic stability will govern both potential practical applications of homopolymers and copolymers.

## 6. Conclusions

The FRP of the double-charged monomers di-M and di-A in aqueous solution revealed nonideality. Several factors including Coulomb interaction, monomer association, steric effects, and specific features of the monomer constitution, all seem to contribute to the nonideality of the kinetic polymerization scheme, to the unusually high monomer reaction orders as well as the observed autoacceleration.

Considering that this was the first detailed study on di-M and di-A and on the homopolymerization of this type of double-charged monomers, a comprehensive kinetic-mechanistic polymerization picture could not be expected. Even for simpler monomers, which have been under study for decades and for which the number of publications is abundant, have kinetic schemes of which the details are still being debated.

The data presented here may have an impact on the rethinking of classical kinetic schemes, to what extent these allow for consideration of for example electrostatic or other concentration-dependent effects, which directly or indirectly influence the kinetics. Moreover, the interesting properties of poly(di-M) and poly(di-A) are also encouraging for continuation of studies.

## Figures and Tables

**Table 1:** Experimental conditions of the batch polymerizations of di-M, di-A, and Q9.

Series	[M] (mol/L)	[I] × 10^2^ (mol/L)	*T* (K)	Monomer type
Polymerizations to high conversion				
1	0.3–1.6	1.6	323	di-M
2	0.86–1.35	1.6	323	di-A
3	0.1–0.6	0.49	323	Q9
Effect of the ionic strength, addition of 1 mol/L NaCl				
4	1.35	1.6	323	di-M
Polymerizations to low conversion				
5	0.3–1.6	1.6	323	di-M
6	0.85–1.23	1.6	323	di-A
7	0.1–0.7	0.49	323	Q9
Effect of the reaction temperature				
8	0.3–1.47	1.6	323–338	di-M
9	1.33	1.6	318–328	di-A
Evaluation of side reactions				
10	1.2–1.5	1.65	323	di-M

**Table 2:** Partial specific volume, $\bar{\nu }$, and partial molar volume, $\bar{V}$ , of di-M, di-A, and Q9 in water at (293 ± 0.01) K; comparison of experimental $\left({\bar{\nu }}_{\text{exp}},\;{\bar{V}}_{\text{exp}}\right)$ and calculated (${\bar{\nu }}_{\text{cal}},\;{\bar{V}}_{\text{cal}})$ values.

Monomer	Molar mass	${\bar{\nu }}_{\text{exp}}$	${\bar{\nu }}_{\text{calc}}$	${\bar{V}}_{\text{exp}}$	${\bar{V}}_{\text{calc}}$	Δ$\bar{V}$
	(g/mol)	(mL/g)	(mL/g)	(mL/mol)	(mL/mol)	(%)
di-M	315.28	0.826	0.785	260.4	247.5	5.0
di-A	301.26	0.807	0.768	243.1	231.6	4.7
Q9	193.67	0.843	0.805	163.3	156.0	4.5

**Table 3:** Intrinsic viscosity, [η], number average molar mass, *M_n_*, and degree of polymerization, *P_n_*, of poly(di-M) samples synthesized at *T* = 323 K, [I] = 1.6 × 10^−2^ mol/L.

Reaction time (min)	Conversion (%)	[η] (mL/g)	*M_n_* (kg/mol)	*P_n_*
[di-M]_0_ = 1.35 mol/L				
25	11.4	37	69.0	219
40	21.1	44	82.6	262
50	24.3	48	90.5	287
62	41.1	54	102.5	325
[di-M]_0_ = 1.45 mol/L				
20	10.5	40	74.7	237
35	21.8	53	99.9	317
45	32.7	64	122.0	387
50	39.8	70	134.0	425

**Table 4:** Intrinsic viscosity, [η], number average molar mass, *M*_n_, and degree of polymerization, *P*_n_, of poly(di-M) samples synthesized with addition of [NaCl] = 1 mol/L at [di-M]_0_ = 1.35 mol/L, *T* = 323 K, [I] = 1.6 × 10^−2^ mol/L.

Reaction time (min)	Conversion (%)	[η] (mL/g)	*M_n_* (kg/mol)	*P_n_*
25	8.3	35	64.9	206
40	14.4	38	70.9	225
50	20.6	40	74.7	237
65	31.1	46	86.7	275

**Table 5:** Overall activation energy, *E*_a_, and frequency factor, ln *A*, for di-M and di-A polymerizations at 318 < *T* < 333 K.

Series	[di-M]_0_ (mol/L)	[di-A]_0_ (mol/L)	*E*_a_ (kJ/mol)	ln *A*
8	1.47	-	20.6	−0.9
8	1.33	-	31.5	4.0
9	-	1.33	103	29

**Table 6:** Intrinsic viscosity [η] of poly(di-M) and poly(di-A) according to Schulz-Blaschke [25] (SB) and Huggins [24] (H) and number average molar mass *M*_n_, in 0.5 mol/L NaCl, *T* = 293 K.

[η]_SB_ (mL/g)	*k*_SB_	[η]_H_ (mL/g)	*k*_H_	*M*_n_ × 10^−3^ (g/mol) ^a^
41	0.44	41	0.49	85.6
59	0.46	58	0.53	107
62	0.38	61	0.44	118
96	0.27	95	0.31	200
239 ^b^	0.27	233	0.41	-
236 ^c^	0.28	229	0.42	-

^a^ by membrane osmometry; ^b^ polymerization at the solubility limit of di-M; ^c^ polymerization at the solubility limit of di-A.

## Supplementary Materials

Supplementary Materials can be found at www.mdpi.com/2073-4360/8/6/234/s1.

## Abbreviations

The following abbreviations are used in this manuscript:

PEL	polyelectrolyte(s)
di-M	1,3-bis(*N,N,N*-trimethylammonium)-2-propylmethacrylate dichloride
di-A	1,3-bis(*N,N,N*-trimethylammonium)-2-propylacrylate dichloride
Q9	acryloyloxyethyltrimethylamonium chloride
DADMAC	diallyldimethylammonium chloride
AMPHC	2,2′-azobis(2-methylpropionamidine) dihydrochloride
[M]	monomer concentration
[I]	initiator concentration
H	Huggins
SB	Schulz-Blaschke
PRT	primary radical termination
DCT	degradative chain transfer
FRP	free radical polymerization
HPLC	high-performance liquid chromatography
SEC	size exclusion chromatography
EPR	electron paramagnetic resonance

## References

[1] Jaeger W., Bohrisch J., Laschewsky A. (2010). Synthetic polymers with quaternary nitrogen atoms-Synthesis and structure of the most used type of cationic polyelectrolytes. Progr. Polym. Sci..

[2] Wandrey C., Hernandez-Barajas J., Hunkeler D. (1999). Diallyldimethylammonium chloride and its polymers. Adv. Polym. Sci..

[3] Matsumoto A., Mano H., Oiwa M. (1989). Polymerization of tetraallyl ammonium chloride. J. Polym. Sci. A.

[4] Hunkeler D., Hamielec E. (1991). Kinetics and modeling of inverse-microsuspension polymerization: 2. Copolymerization of acrylamide with quaternary ammonium cationic monomers. Polymer.

[5] Beuermann S., Paquest D.A., McMinn J.H., Hutchinson R.A. (1997). Propagation kinetics of methacrylic acid studied by pulsed laser polymerization. Macromolecules.

[6] Beuermann S., Buback M., Hesse P., Lacík I. (2006). Free-radical propagation rate coefficient of nonionized methacrylic acid in aqueous solution from low monomer concentrations to bulk polymerization. Macromolecules.

[7] Beuermann S., Buback M., Hesse P., Kuchta F.-D., Lacík I., van Herk A.M. (2007). Critically evaluated rate coefficients for free-radical polymerization. 6. Propagation rate coefficient for methacrylic acid. Pure Appl. Chem..

[8] Beuermann S., Buback M., Hesse P., Kukučková S., Lacík I. (2007). Propagation rate coefficient of non-ionized methacrylic acid radical polymerization in aqueous solution. The effect of monomer conversion. Macromol. Symp..

[9] Beuermann S., Buback M., Hesse P., Hutchinson R.A., Kukučková S., Lacík I. (2008). Termination kinetics of the free-radical polymerization of non-ionized methacrylic acid in aqueous solution. Macromolecules.

[10] Buback M., Hesse P., Hutchinson R.A., Kasák P., Lacík I., Stach M., Utz I. (2008). Kinetics and modeling of free-radical batch polymerization of nonionized methacrylic acid in aqueous solution. Ind. Eng. Chem. Res..

[11] Wittenberg N.F.G., Buback M., Hutchinson R.A. (2013). Kinetics and modeling of methacrylic acid radical polymerization in aqueous solution. Macromol. React. Eng..

[12] Wittenberg N.F.G., Preusser C., Kattner H., Stach M., Lacík I., Hutchinson R.A., Buback M. (2015). Modeling acrylic acid radical polymerization in aqueous solution. Macromol. React. Eng..

[13] Lacík I., Beuermann S., Buback M. (2004). PLP-SEC study into free-radical propagation rate coefficients of partially and fully ionized acrylic acid in aqueous solution. Macromol. Chem. Phys..

[14] Beuermann S., Buback M., Hesse P., Kukučková S., Lacík I. (2007). Propagation kinetics of Free-radical methacrylic acid polymerization in aqueous solution. the effect of concentration and degree of ionization. Macromol. Symp..

[15] Lacík I., Učňová L., Kukučková S., Buback M., Hesse P., Beuermann S. (2009). Propagation rate coefficient of free-radical polymerization of partially and fully ionized methacrylic acid in aqueous solution. Macromolecules.

[16] Barth J., Buback M. (2012). Termination and transfer kinetics of sodium acrylate polymerization in aqueous solution. Macromolecules.

[17] Beuermann S., Buback M., Hesse P., Junkers T., Lacík I. (2006). Free-radical polymerization kinetics of 2-acrylamido-2-methylpropanesulfonic acid in aqueous solution. Macromolecules.

[18] Okayasu T., Hibino T., Nishide H. (2011). Free radical polymerization kinetics of vinylsulfonic acid and highly acidic properties of its polymer. Macromol. Chem. Phys..

[19] Buback M., Feldermann A., Kowollik C., Lacík I. (2001). Propagation rate coefficients of Acrylate-Methacrylate free-radical bulk copolymerizations. Macromolecules.

[20] Beuermann S., Buback M. (2002). Rate coefficients of free-radical polymerization deduced from pulsed laser experiments. Progr. Polym. Sci..

[21] Losada R., Wandrey C. (2008). Non-ideal polymerization kinetics of a cationic double charged acryl monomer and solution behavior of the resulting polyelectrolyte. Macromol. Rapid Commun..

[22] Losada R., Wandrey C. (2009). Copolymerization of a cationic double-charged monomer and electrochemical properties of the copolymers. Macromolecules.

[23] Vanneste P., Loenders R., Vanden Eynde I., Eeckhaoudt S. (2005). Preparation of (Meth)Acrylate Diammonium Salts and Their Use as Monomers for The Synthesis of Polymers. Patent.

[24] Huggins M.L. (1942). Theory of solution of high polymers. J. Am. Chem. Soc..

[25] Schulz G.V., Blaschke F.J. (1941). Molecular-weight determinations on macromolecular materials. IX. An equation for the calculation of the viscosity number at very small concentrations. Prakt. Chem..

[26] Wandrey C., Hunkeler D., Tripathy S.K., Kumar J., Nalva H.S. (2002). Handbook of Polyelectrolytes and their Applications.

[27] Wandrey C. (1997). Polyelektrolyte-Makromolekulare Parameter und Elektrolytverhalten.

[28] Odian G. (2004). Principles of Polymerization.

[29] Mayo F.R. (1943). Chain transfer in the polymerization of styrene: The reaction of solvents with free radicals. J. Am. Chem. Soc..

[30] Trommsdorff V.E., Köhle H., Lagally P. (1948). Zur Polymerisation des methacrylsäuremethylesters. Macromol. Chem..

[31] Balke S.T., Hamielec A.E. (1973). Bulk polymerization of methyl methacrylate. J. Appl. Polym. Sci..

[32] Turner D.T. (1977). Autoacceleration of free radical polymerization. 1. The critical concentration. Macromolecules.

[33] Small P.A. (1975). Long-chain branching in polymers. Adv. Polym. Sci..

[34] O’Neill G.A., Wisnudel M.B., Torkelson J.M. (1998). Gel effect in free radical polymerization: Model discrimination of its cause. AIChE J..

[35] Odian G. (2004). Principles of Polymerization.

[36] O’Neill G.A., Wisnudel M.B., Torkelson J.M. (1998). An evaluation of free volume approaches to describe the gel effect in free radical polymerization. Macromolecules.

[37] Kohonen M.M., Karaman M.E., Pashley R.M. (2000). Debye length in multivalent electrolyte solutions. Langmuir.

[38] Tadmor R., Hernandez-Zapata E., Chen N., Pincus P., Israelachvili J.N. (2002). Debye length and double-layer forces in polyelectrolyte solutions. Macromolecules.

[39] Dobrynin A.V., Rubinstein M. (2005). Theory of polyelectrolytes in solution and at surfaces. Prog. Polym. Sci..

[40] Kékicheff P., Ninham B.W. (1990). The double-layer interaction in asymmetric electrolytes. Europhys. Lett..

[41] Manning G.S. (1969). Limiting laws and counterion condensation in polyelectrolyte solutions I. Colligative properties. J. Chem. Phys..

[42] Durchschlag H., Zipper P. (1994). Calculation of the partial volume of organic compounds and polymers. Progr. Colloid Polym. Sci..

[43] Durchschlag H., Zipper P. (1997). Calculation of partial specific volumes and other volumetric properties of small molecules and polymers. J. Appl. Crystallogr..

[44] Brandrup J., Immergut E.H. (1999). Polymer Handbook.

[45] Wako Pure Chemical Ind., Ltd. Wako Chemicals Information Brochure. http://www.wako-chem.co.jp/english/labchem/index_chemical.htm.

[46] Manning G.S. (1965). Cluster theory of polyelectrolyte solutions. I. Activity coefficients of the mobile ions. J. Chem. Phys..

[47] Manning G.S. (1969). Limiting laws and counterion condensation in polyelectrolyte solutions II. Self-diffusion of the small ions. J. Chem. Phys..

[48] Gueron M., Weisbuch G.J. (1979). Polyelectrolyte theory. 2. Activity coefficients in Poisson-Boltzmann and in condensation theory of the counterion sheet. J. Chem. Phys..

[49] Zhang H.-L., Han S.-J. (1996). Viscosity and density of Water + Sodium Chloride + Potassium chloride solutions at 298.15 K. J. Chem. Eng. Data.

[50] Yu Y.-X., Gao G.-H., Li Y.-G. (2000). Surface tension for aqueous electrolyte solutions by the modified mean spherical approximation. Fluid Ph. Equilib..

[51] Sato T., Hashimoto M., Seno M., Hirano T. (2003). Elementary reaction analysis of the radical polymerization of -ethyl-*N*-(-methylbenzyl)itaconamates carrying (RS)- and (S)-methylbenzylaminocarbonyl groups. J. Polym. Sci. Part A Polym. Chem..

[52] Seno M., Fukui T., Hirano T., Sato T. (2000). Kinetic and ESR studies on radical polymerization behavior of *N*-(2-phenylethoxycarbonyl)methacrylamide. J. Polym. Sci. Part A.

[53] Gromov V.F., Galperina N.I., Osmanov T.O., Khomikovskii P.M., Abkin A.D. (1980). Effect of solvent on chain propagation and termination reaction rates in radical polymerisation. Eur. Polym. J..

[54] Wang H., Miyamoto A., Hirano T., Seno M., Sato T. (2004). Radical polymerization of 2-methacryloyloxyethyl phosphorylcholine in water: Kinetics and salt effects. Eur. Polym. J..

[55] Gromov V.F., Bogachev Yu. S., Bune Y.V., Zhuravleka I.L., Teleshov E.N. (1991). Radical polymerization of water-soluble monomers in various solvents. Eur. Polym. J..

[56] Heuts J.P.A., Gilbert R.G., Radom L.A. (1995). Priori prediction of propagation rate coefficients in free-radical polymerizations: Propagation of ethylene. Macromolecules.

[57] Suleimenov I.E., Rustemova E.M., Bekturov E.A. (2007). Mechanisms of viscosity of polyacids and polybases in the region of pronounced polyelectrolyte effect. J. Polym. Sci. A.

[58] De Kock J.B.L., van Herk A.M., German A.L. (2001). Bimolecular free-radical termination at low conversion. J. Macromol. Sci. Polym. Rev..

[59] Wandrey C. (1980). Zur Bruttokinetik der Cyclopolymerisation von Dimethyl-diallyl-ammoniumchlorid. Ph.D. Thesis.

[60] Deb P.C., Meyerhoff G. (1974). Primary radical termination in polymerization: Evaluation of the characteristic constant. Eur. Polym. J..

[61] Deb P.C. (1975). Nonideal polymerization. Treatment of nonideality due to primary radical termination and degradative chain transfer. Eur. Polym. J..

[62] Ghosh P., Banerjee A.N., Mitra P.S., Chakraborty S. (1975). Chain transfer in presence of halogens in polymerization of methylmethacrylate. J. Polym. Sci. Part C.

[63] Shukla P., Srivastava A.K. (1988). Radical polymerization of methyl methacrylate initiated by pyridine dicyanomethylide. Eur. Polym. J..

[64] Brandrup J., Immergut E.H. (1999). Polymer Handbook.

[65] Topchiev D.A., Nashmetdinova G.T. (1983). Kinetics of radical polymerization of *N*,*N*-dialkyl-*N*,*N*-diallylammonium chlorides. Vysokomol. Soed. A.

[66] Wandrey C., Jaeger W., Reinisch G. (1981). Zur Kinetik der radikalischen Polymerisation von Dimethyl-dially-lammoniumchlorid. I. Bruttokinetik bei niedrigen Umsätzen und Versuche zu ihrer Deutung. Acta Polym..

[67] Hahn M., Jaeger W., Wandrey C., Seehaus F., Reinisch G. (1984). Cyclopolymerization kinetics of dimethyldiallylammonium chloride. J. Macromol. Sci. Chem. A.

[68] Pascal P., Napper D.H., Gilbert R.G., Piton M.C., Winnik M.A. (1990). Pulsed laser study of the propagation kinetics of acrylamide and methacrylamide in water. Macromolecules.

[69] Kukulj D., Davis T.P. (1998). Average propagation rate coefficients in the free-radical copolymerization of styrene and α-methylstyrene measured by pulsed-laser polymerization. Macromolecules.

[70] Morris L.M., Davis T.P., Chaplin R.P. (2001). Radical copolymerization propagation kinetics of methyl ethylacrylate and styrene. Polymer.

[71] Gilbert B.C., Smith J.R.L., Milne E.C., Whitwood A.C., Taylor P. (1994). Kinetic and structural EPR studies of radical polymerization. Monomer, dimer, trimer and mid-chain radicals formed via the initiation of polymerization of acrylic acid and related compounds with electrophilic radicals (^·^OH, SO_4_^−·^ and Cl_2_^−·^). J. Chem. Soc. Perkin Trans..

[72] Buback M., Hesse P., Lacík I. (2007). Propagation rate coefficient and fraction of mid-chain radicals for acrylic acid polymerization in aqueous solution. Macromol. Rapid Commun..

[73] Manning G.S., Mohanty U. (1997). Counterion condensation on ionic oligomers. Phys. A.

[74] Lacík I., Beuermann S., Buback M. (2001). Aqueous phase size-exclusion-chromatography used for PLP-SEC studies into free-radical propagation rate of acrylic acid in aqueous solution. Macromolecules.

[75] Lacík I., Beuermann S., Buback M. (2003). PLP-SEC study into free-radical propagation rate of non-ionized acrylic acid in aqueous solution. Macromolecules.

[76] Buback M., Egorov M., Junkers T., Panchenko E. (2004). Free-radical termination kinetics studied using a novel SP–PLP–ESR technique. Macromol. Rapid Commun..

[77] Barth J., Buback M. (2010). SP-PLP-EPR—A novel method for detailed studies into the termination kinetics of radical polymerization. Macromol. React. Eng..

[78] Barth J., Buback M. (2011). SP-PLP-EPR Study into the termination kinetics of methacrylic acid radical polymerization in aqueous solution. Macromolecules.

[79] Barth J., Meiser W., Buback M. (2012). SP-PLP-EPR study into termination and transfer kinetics of non-ionized acrylic acid polymerized in aqueous solution. Macromolecules.

[80] Owenius R., Trry G.E., Williams M.J., Eaton S.S., Eaton G.R. (2004). Frequency dependence of electron spin relaxation of Nitroxyl radicals in fluid solution. J. Phys. Chem. B.

[81] Yamada B., Sugiyama S., Mori S., Otsu T. (1981). Low ceiling temperature in radical polymerization of 2,6-dimethylphenyl methacrylate. J. Macromol. Sci. Chem..

[82] Penelle J., Xie T. (2001). Synthesis, characterization and thermal properties of poly(trimethylene-1,1-dicarboxylate) polyelectrolyte. Macromolecules.

