# Supplementary material for: Effect of Very High Charge Density and Monomer Constitution on the Synthesis and Properties of Cationic Polyelectrolytes
Source: Polymers (Basel). 2016 Jun 16;8(6):234. doi: 10.3390/polym8060234 (PMC6432479; doi:10.3390/polym8060234)
Supplement: Supplementary file 1 [file polymers-08-00234-s001.pdf]

Supplementary Materials: Effect of Very High Charge Density and Monomer Constitution on the Synthesis and Properties of Cationic Polyelectrolytes

Hamideh Ahmadloo, Ricardo Losada and Christine Wandrey

1. Polyelectrolyte Synthesis

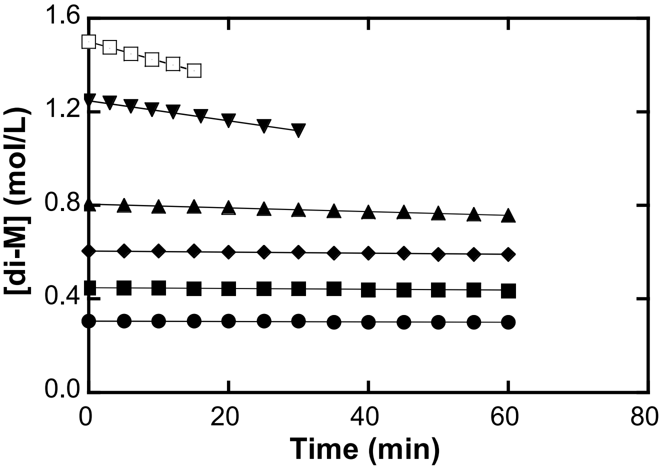

Figure S1. Influence of the initial monomer concentration $[di\text{-}M]_0$ on the linear period of $-d[M]/dt$. $[di\text{-}M]_0$ = ●0.3, ■0.45, ♦0.6, ▲0.8, ▼1.25, □1.5 mol/L, [I] = 1.6×10^{-2} mol/L, T = 323 K.

2. Permanently Charged Polyelectrolytes in Solution

Calculation of the Bjerrum length l_B according to Equation (8):
with $e = 1.60218 \times 10^{-19}$ C; $\varepsilon_0 = 8.8542 \times 10^{-12}$ $s^4 \cdot A^2/m^3 \cdot kg$; $\varepsilon_{0,\ water,\ 293\ K} = 80.1$; $\varepsilon_{0,\ water,\ 298\ K} = 78.3$;
$k_B = 1.3807 \times 10^{-23}$ J/K;
l_B = 0.712 nm in water at 293 K
l_B = 0.714 nm in water at 298 K

Calculation of the theoretical/structural charge spacing b (Scheme III):
b = monomer unit length/number of charges
for di-M and di-A: b = 0.25/2 = 0.125 nm (C–C bond length 0.154 nm, bond angle 112°)
for Q9: b = 0.25/1 = 0.25 nm

3. Monomer and Monomer Solution Properties

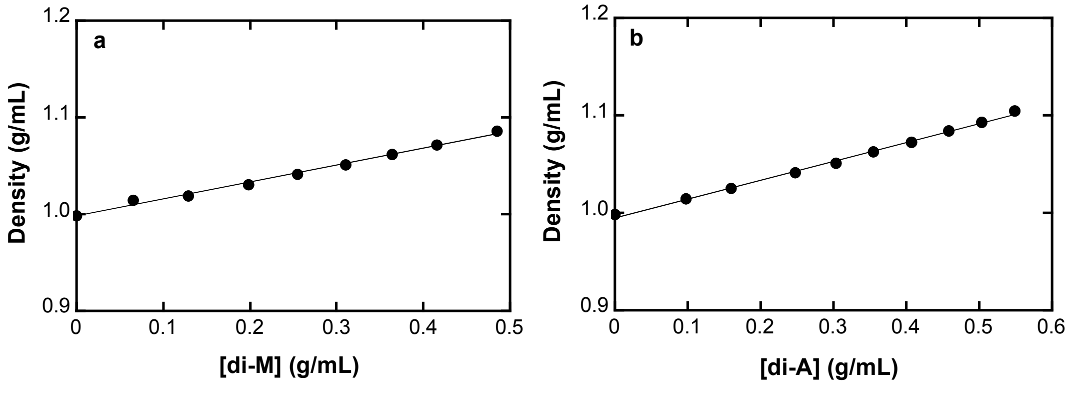

Figure S2. *Cont.*

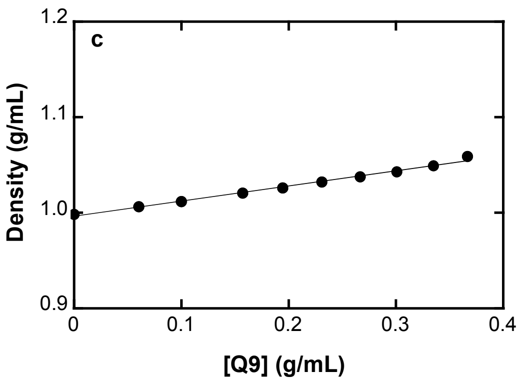

Figure S2. Influence of the monomer concentration on the density of aqueous monomer solutions of (**a**) di-M; (**b**) di-A; (**c**) Q9, $T = (293 \pm 0.01)$ K.

4. Polyelectrolyte and Polyelectrolyte Solution Properties

4.1. Intrinsic Viscosity and Molar Mass

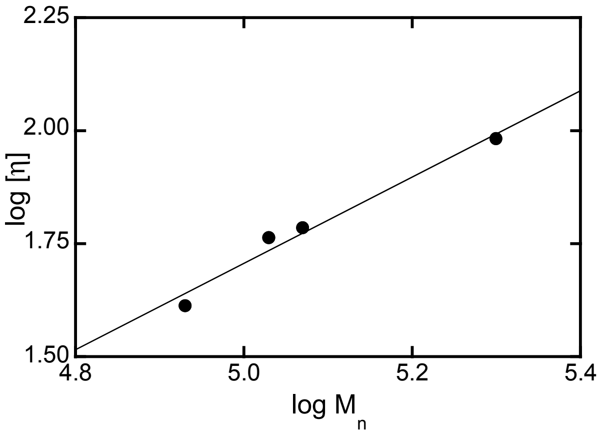

Figure S3. Determination of the intrinsic viscosity-molar mass relationship for poly(di-M) in 0.5 mol/L NaCl, $T = 293$ K, $8 \times 10^4 < M_n < 2 \times 10^5$ g/mol, by membrane osmometry.

4.2. Counterion Condensation

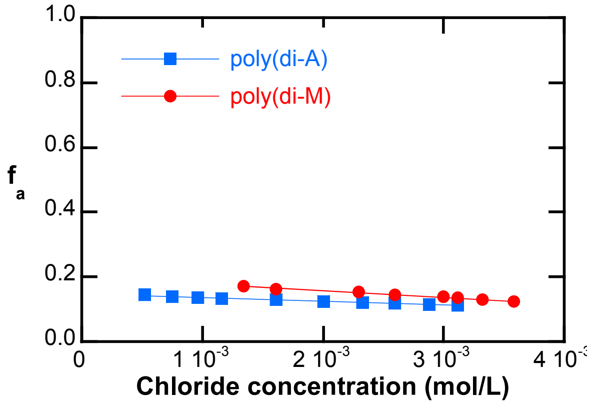

Figure S4. Counterion activity coefficients f_a of poly(di-A) and poly(di-M) dissolved in water, $T = 293$ K.

5. Autoacceleration

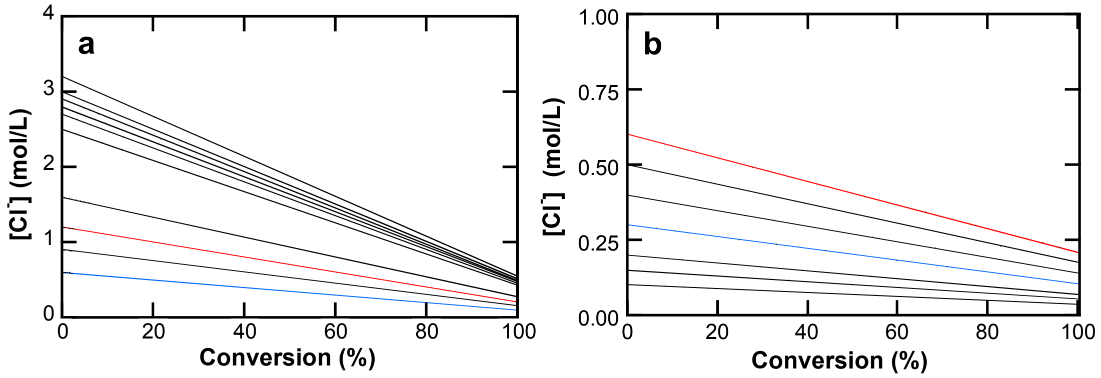

Figure S5. Decrease of the free chloride ion concentration [Cl⁻] *vs.* conversion (**a**) di-M and di-A: 0.3 < $[M]_0$ < 1.6 mol/L; (**b**) Q9: 0.1 < $[M]_0$ < 0.6 mol/L. The red and blue lines indicate the same initial monomer concentrations 0.6 and 0.3 mol/L.

Equation (12) was used to quantify the decrease of the ionic strength expressed as the decrease of the concentration of the free chloride ions [Cl⁻].

$$[\mathrm{Cl}^-] = n\{[\mathrm{M}] + [\mathrm{P}](1/\xi)\} = n\{[\mathrm{M}]_0(1 - X_\mathrm{M}) + X_\mathrm{M}(1/\xi)\} \quad (12)$$

with n the number of charges per monomer molecule, n = 2 for di-M and di-A, but n = 1 for Q9. [M] and [P] are the monomer and polymer concentrations, X_M is the monomer conversion at time t, and ξ is the Manning charge-density parameter defined in Equation (10).

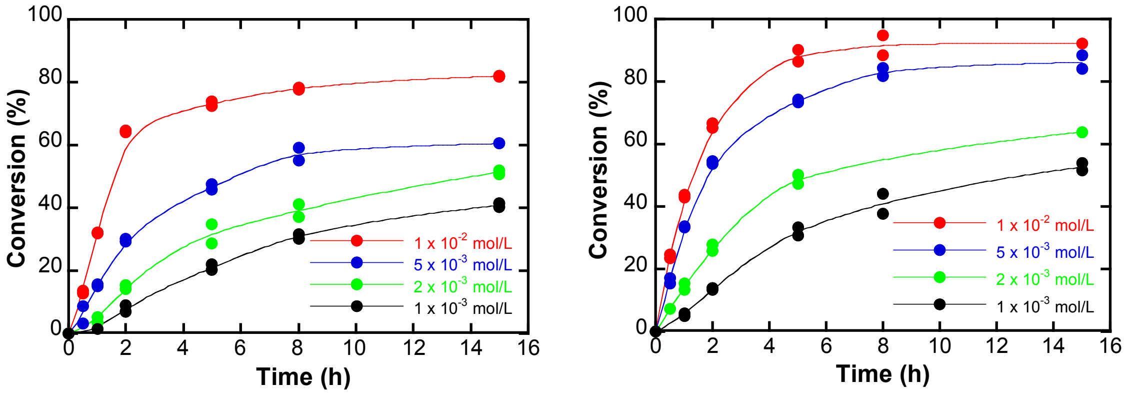

(**a**) (**b**)

Figure S6. Conversion curves of ammonium persulfate-initiated free radical polymerizations of DADMAC in aqueous solution at T = 323 K, 1.0 × 10^{-3} < [I] < 1.0 × 10^{-2} mol/L (**a**) $[\mathrm{DADMAC}]_0$ = 3.0 mol/L; (**b**) $[\mathrm{DADMAC}]_0$ = 4.0 mol/L.

© 2016 by the authors; licensee MDPI, Basel, Switzerland. This article is an open access article distributed under the terms and conditions of the Creative Commons Attribution (CC-BY) license (http://creativecommons.org/licenses/by/4.0/).
